# Differences in immune-related semaphorin levels in the CSF and serum of newly diagnosed, treatment-naive patients with relapsing–remitting multiple sclerosis: a case–control study

**DOI:** 10.3389/fneur.2026.1851383

**Published:** 2026-07-14

**Authors:** Furkan Sarıdaş, Birnur Aydın, Rifat Özpar, Emine Rabia Koç, Bahattin Hakyemez, Tülin Alkan, Ömer Faruk Turan

**Affiliations:** 1 Department of Neurology, Bursa Uludağ University Medicine Faculty, Bursa, Türkiye; 2 Department of Physiology, Bursa Uludağ University Medicine Faculty, Bursa, Türkiye; 3 Department of Radiology, Bursa Uludağ University Medicine Faculty, Bursa, Türkiye

**Keywords:** cerebrospinal fluid (CSF), immune semaphorins, oligoclonal bands (OCBs), relapsing–remitting multiple sclerosis (RRMS), semaphorin 3A (SEMA3A), semaphorin 4A (SEMA4A), semaphorin 4D (SEMA4D), semaphorin 7A (Sema7A)

## Abstract

**Background:**

The pathogenesis of multiple sclerosis (MS) remains incompletely elucidated. Semaphorins play an active role in the immune and nervous systems by regulating receptor-mediated adhesion mechanisms. Immunosemaphorins have recently emerged as diagnostic biomarkers or therapeutic targets in MS.

**Objectives:**

This study aimed to investigate alterations in semaphorins (Sema) 3A, 3F, 4A, 4D, and 7A in newly diagnosed, treatment-naive patients with relapsing–remitting multiple sclerosis (RRMS).

**Methods:**

In this prospective case–control study, semaphorin levels were evaluated by enzyme-linked immunosorbent assay in 40 consecutive patients (serum and cerebrospinal fluid (CSF)) and 40 healthy controls (serum).

**Results:**

Serum levels of Sema3A, Sema4A, Sema4D, and Sema7A were lower in RRMS patients than in healthy controls and remained significant after correction for multiple testing. In exploratory analyses, OCB count was negatively correlated with serum Sema4A and Sema7A and positively correlated with CSF Sema7A. Patients with an OCB count ≥12 had lower serum levels of Sema3F, Sema4A, and Sema7A. Sema4D levels were higher in patients with an IgG index ≥0.7. Patients with infratentorial lesions had lower serum Sema4A levels and higher CSF Sema4A levels.

**Conclusion:**

Low serum levels of Sema3A may be diagnostically relevant in RRMS. Low serum levels of Sema7A and elevated CSF levels may be associated with inflammatory immune activation. Further studies are needed for Sema3F, Sema4A, and Sema4D.

## Introduction

Multiple sclerosis (MS) is a chronic immune-mediated, demyelinating, neuroinflammatory, and neurodegenerative disease of the central nervous system (CNS), characterized by immune cell infiltration, blood–brain barrier disruption, myelin loss, and progressive neuronal damage (1). Although its exact neuroimmunological pathogenesis remains incompletely understood, accumulating evidence suggests that immune-regulatory molecules may contribute to disease development and progression.

Semaphorins are a large family of secreted and membrane-associated signaling proteins initially identified as axon guidance molecules but now recognized as important regulators of both the nervous and immune systems (2, 3). In recent years, several semaphorin family members, particularly Sema3A, Sema3F, Sema4A, Sema4D, and Sema7A, have attracted attention because of their potential roles in autoimmunity and neuroinflammation (4–6). However, most available data are derived from experimental models, and clinical studies in MS remain limited. Class 3 semaphorins are involved in intercellular communication, axon guidance, and immune regulation (4). Sema3A suppresses dendritic cell migration, T-cell proliferation, and proinflammatory cytokine production while enhancing regulatory immune functions. In addition, Sema3A and Sema3F have been associated with oligodendrocyte progenitor cell recruitment and lesion remyelination in MS and demyelination models (7–13). Sema4A is expressed in dendritic cells, B cells, and T-helper cells in lymphoid tissues and plays an important role in T-cell activation, IL-2 expression, and Th1, Th2, and Th17 differentiation (6, 14–17). Experimental studies suggest that Sema4A contributes to the Th-mediated pathogenesis of MS and EAE (experimental autoimmune encephalomyelitis), as Sema4A deficiency confers resistance to EAE and anti-Sema4A monoclonal antibody treatment ameliorates disease severity (4, 15, 18, 19). Sema4D is expressed on T cells, activated B cells, mature dendritic cells, macrophages, and neutrophils, and is involved in immune activation through regulation of B-cell and T-cell responses (20–23). It has also been implicated in axonal growth and blood–brain barrier integrity, acting together with Sema3A in these processes (6, 17, 24). In experimental models, Sema3F promotes remyelination, whereas Sema3A and Sema4D inhibit it (6). Moreover, Sema4D has been associated with microglial activation, disruption of endothelial tight junctions, neuroinflammation, and impaired myelination in EAE, while anti-Sema4D treatment reduces inflammation and improves remyelination, suggesting potential therapeutic relevance in MS (4, 19, 24–32). Among class 7 semaphorins, Sema7A is the only immune semaphorin and acts as a positive regulator of immune responses through α1β1 integrin-mediated signaling, promoting macrophage activation and proinflammatory cytokine production (26, 33, 34). In EAE, increased Sema7A expression has been associated with immune modulation, neuroinflammation, and neurodegeneration, whereas Sema7A deficiency confers resistance to disease development and may protect against demyelination (35, 36). These findings suggest that Sema7A and α1β1 integrin interactions may represent potential therapeutic targets in MS (4, 33).

In light of these findings, this study aimed to investigate the associations of semaphorins 3A, 3F, 4A, 4D, and 7A with disease development and clinical and radiological characteristics in treatment-naive patients with multiple sclerosis.

## Materials and methods

### Setting up patient and control groups

The study was approved by the Bursa Uludağ University Clinical Research Ethics Committee (approval no. 2022-16/36; date: 14 November 2022). Written informed consent was obtained from all participants. Forty consecutive patients, aged 18–65 years, with a definite diagnosis of RRMS according to the revised 2017 McDonald criteria, and serum and cerebrospinal fluid (CSF) samples obtained at the Bursa Uludag University Faculty of Medicine, Department of Neurology and 40 consecutive healthy controls (HC) with age- and sex-matched serum samples, without past or current neurological complaints, were included in the study. Patients with other neurological or immune system disorders and those with an active infection during the study were excluded.

### Demographic, clinical, and radiological assessment

Demographic data, initial symptoms, number of symptoms, neurological examination, Expanded Disability Status Scale (EDSS), and neuropsychometric tests [Symbol Digit Modalities Test (SDMT) and Montreal Cognitive Assessment (MoCA) tests] were recorded by neurologists. The number and location of periventricular (FLAIR), juxtacortical/cortical (FLAIR), infratentorial (T2W), and spinal (T2W) lesions were determined by two independent, blinded neuroradiologists according to Barkhof-Tintore MR imaging criteria.

### Biospecimen collection and ELISA analysis

Blood samples collected by phlebotomy from the study and control groups were placed in empty tubes without gel, allowed to stand for 15–20 min, centrifuged at 30,000 rpm for 20 min, and the serum portion was separated. CSF samples were transferred from 2 mL sterile gel-free tubes obtained from the study group to 1 mL Eppendorf tubes by lumbar puncture in the lateral decubitus position. All samples were numbered, anonymized, and stored in a cold chain at −80 °C. They were then analyzed spectrophotometrically by a blinded investigator using a specific enzyme-linked immunosorbent assay kit (BT LAB, Shanghai, China) according to the manufacturer’s instructions and in line with previously published studies in patients with multiple sclerosis (37, 38). All samples were analyzed in duplicate, and the mean value of duplicate measurements was used for statistical analysis. Samples showing poor agreement between duplicate wells, defined as a coefficient of variation (CV) > 20%, were excluded from the final analyses as part of the assay quality control procedure. Consequently, the number of analyzed samples differed slightly between biomarkers. The measurements were performed using a spectrophotometer (BioTek Quant, BioTek Instruments Inc., USA) at a wavelength of 450 nm. Concentrations were calculated from standard curves generated for each assay plate and expressed as ng/mL.

To assess assay reproducibility, intra-assay coefficients of variation were calculated from duplicate measurements. The mean intra-assay CVs were 2.48% for SEMA3A, 6.47% for SEMA3F, 6.36% for SEMA4A, 4.07% for SEMA4D, and 7.36% for SEMA7A. Overall, across all analytes and sample groups, the mean intra-assay CV was 5.36% (median 3.27%), indicating acceptable assay precision. To minimize potential matrix-related variability between serum and CSF samples, all specimens were processed under identical preanalytical and analytical conditions.

### Statistical analysis

Demographic data, clinical features, and radiological evaluations of the patients were analyzed using IBM SPSS Statistics 29.0.2.0 (IBM Corp., 2023). Data normality was assessed using the Shapiro–Wilk test. Descriptive statistics were determined as mean and standard deviation or median (minimum–maximum) for quantitative data. One-way analysis of variance was used to compare more than two groups when the data were normally distributed. Kruskal–Wallis and Mann–Whitney *U* tests were used for non-normally distributed data.

Given the exploratory nature of the study and the number of biomarker-related analyses, the statistical analyses were interpreted according to a predefined hierarchy. The primary analyses consisted of serum case–control comparisons of the five semaphorins between RRMS patients and healthy controls. For these primary comparisons, *p*-values were adjusted for multiple testing across the five serum semaphorins using the Holm–Bonferroni method.

Correlation analyses between semaphorin levels and clinical, cognitive, laboratory, and radiological variables, as well as subgroup comparisons within the RRMS cohort, were considered secondary exploratory analyses. For these exploratory analyses, unadjusted *p*-values are presented to provide full transparency; however, they are interpreted cautiously due to the increased risk of type I error. Findings with *p*-values close to the conventional significance threshold were not considered confirmatory and were interpreted as hypothesis-generating. Where applicable, *post-hoc* pairwise comparisons after significant omnibus tests were adjusted using the Holm–Sidak method. A two-sided *p*-value <0.05 was considered statistically significant for primary analyses after correction, whereas secondary analyses were interpreted descriptively and exploratorily. Pearson’s correlation coefficient was used to analyze relationships when the variables were normally distributed, and Spearman’s rank correlation coefficient when they were not. Graphs were generated using GraphPad Prism, version 9.5.0 (GraphPad Software, Inc., San Diego, CA).

**Figure 1:**
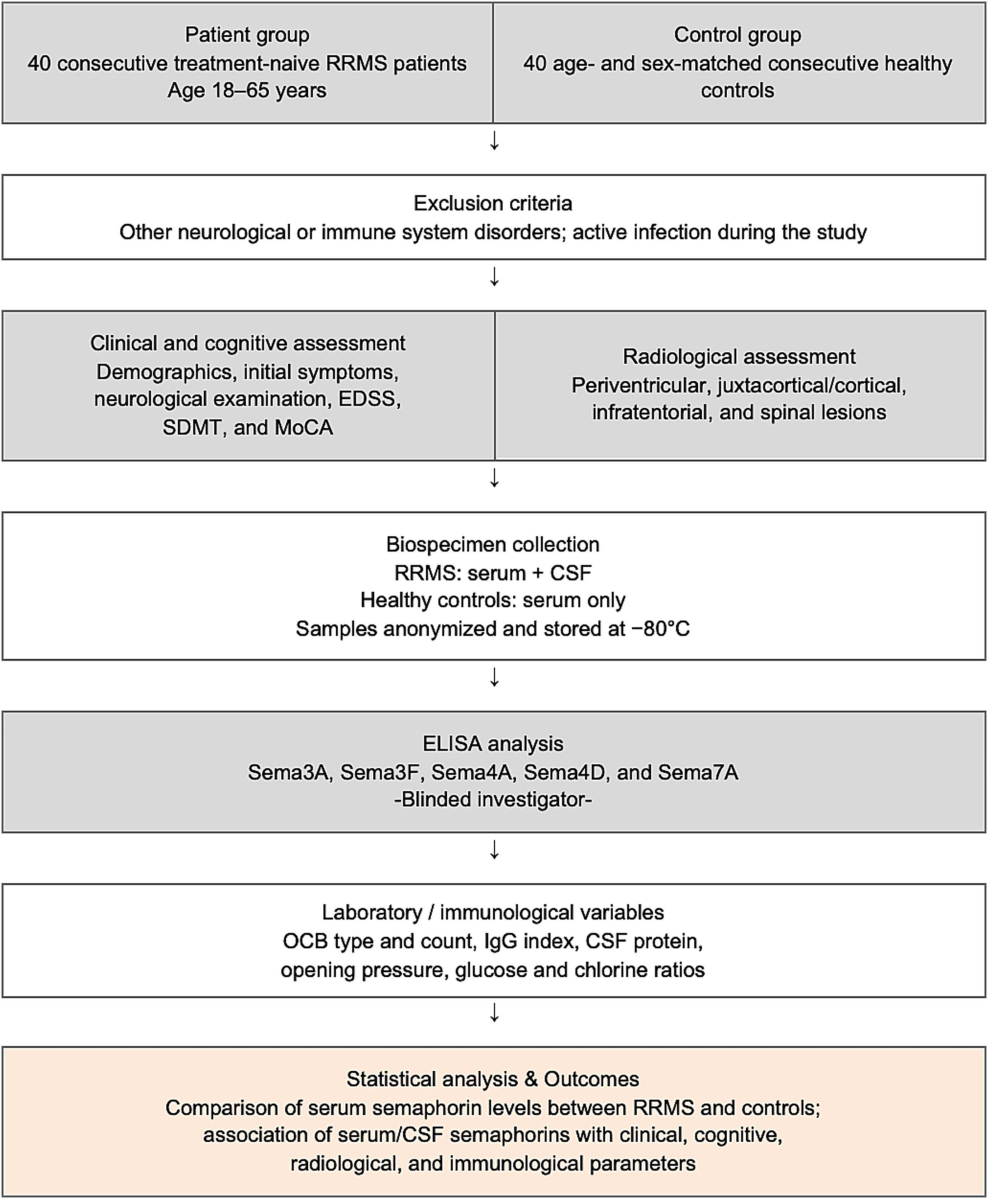
Flow chart of the study design, participant selection, biospecimen collection, ELISA measurements, and outcome assessments. RRMS, relapsing–remitting multiple sclerosis; CSF, cerebrospinal fluid.

Because no formal *a priori* power calculation was performed, the present analyses should be considered exploratory. To aid interpretation of non-significant findings, we performed a sensitivity-based assessment of detectable effect sizes based on the available sample sizes. With a two-sided *α* level of 0.05 and 80% power, the originally recruited case–control cohort of 40 RRMS patients and 40 healthy controls was powered to detect a between-group difference of approximately Cohen’s *d* = 0.63. Because analyte-specific sample sizes varied slightly after assay quality control, the detectable effect size for individual biomarkers was modestly higher; for serum Sema3F, it was approximately Cohen’s *d* = 0.68. Similarly, within the RRMS group, correlation analyses were powered to detect correlations of approximately |*r*| = 0.43–0.45 or larger. Therefore, smaller effects or modest correlations may have remained undetected. A flow chart summarizing the study design and methodological procedures is presented in Figure 1.

## Results

The mean age of the 40 patients (31 women and 9 men) was 33.71 ± 9.98 years. Half of the patients had more than one symptom at the onset of an attack. The most common symptoms were sensory, optic neuritis, brainstem/cerebellar, and motor. Six patients had headaches, three had bladder/bowel symptoms, two had seizures, and one had syncope/presyncope. Half of the patients had at least one poor prognostic initial symptom, including motor, brainstem/cerebellar, or sphincter involvement. The mean initial EDSS score was 1.24 ± 0.90 (range, 1–3). The initial EDSS score was ≥2 in one-third of patients and ≥3 in three patients. Mean CSF protein, opening pressure, CSF/serum glucose ratio, and CSF/serum chloride ratio were within normal limits. The most common OCB pattern was type 2. The OCB count ranged from 3 to 27. The most common lesion location was periventricular, followed by infratentorial involvement. Lesion burden was highest in the periventricular region, followed by the spinal and infratentorial regions (Table 1).

**Table 1:** Clinical, laboratory, and radiological characteristics of RRMS patients.

Features			*n* (%) or mean ± SD (min–max)	Features			*n* (%) or mean ± SD (min–max)
Symptoms and signs at the initial relapse	Optic neuritis		13 (32.5)	Disability and cognitive status		EDSS	1.24 ± 0.9 (1–3)
	Motor		11 (27.5)			EDSS ≥ 2	13 (32.5)
	Sensory		18 (45)			MoCA	23.96 ± 2.96 (17–28)
	Brain stem/cerebellar		12 (30)			SDMT	33.93 ± 15.01 (4–61)
	Other		12 (30)	Radiological analysis	Localization	Cortical/juxtacortical	18 (45)
CSF findings	Opening pressure, cmH₂O		14.48 ± 4.79 (5–24)				
	Protein, mg/dL		30.46 ± 11.64 (10.6–73.9)			Periventricular	38 (95)
	CSF/serum glucose ratio		0.63 ± 0.11 (0.37–0.86)			Infratentorial	27 (67.5)
	CSF/serum chlorine ratio		1.17 ± 0.3 (1.09–1.25)			Spinal cord	14 (35)
	Cell count, /μL		2.27 ± 4.43 (0–20)^*^		Number of plaques	Cortical/juxtacortical	1.35 ± 1.86 (0–8)
	OCB type	Negative	8 (20)			Periventricular	10.72 ± 7.8 (0–29)
		Type 2	25 (62.5)				
		Type 3	7 (17.5)				
	OCB count		12 ± 6.9 (3–27)			Infratentorial	2.07 ± 3.44 (0–16)
	OCB count >12		12 (48)				
	IgG index		0.97 ± 0.56 (0.39–2.77)			Spinal cord	2.87 ± 3.4 (0–13)
	IgG index >0.7		24 (60)				

^*^Mononuclear leukocyte predominance: 74.1% (50–95).

*n*(%) or mean ± SD (min–max). RRMS: relapsing–remitting multiple sclerosis; EDSS: Expanded Disability Status Scale; MoCA: Montreal Cognitive Assessment; SDMT: Symbol Digit Modalities Test; CSF: cerebrospinal fluid; OCB: oligoclonal band; IgG: immunoglobulin G.

Serum semaphorin data were available for 70 participants for Sema3A (37 RRMS patients and 33 controls), 70 for Sema3F (36 RRMS patients and 34 controls), 69 for Sema4A (36 RRMS patients and 33 controls), 71 for Sema4D (37 RRMS patients and 34 controls), and 64 for Sema7A (35 RRMS patients and 29 controls). In CSF, measurements were available for 37 patients for Sema3A and for 40 patients each for Sema3F, Sema4A, Sema4D, and Sema7A. In the overall cohort and across study groups, semaphorin levels did not differ according to sex or age (Supplementary Tables 1, 2). No significant differences in semaphorin levels were observed over time from symptom onset to sample collection (Supplementary Table 3). Serum and CSF semaphorin levels were not significantly correlated in RRMS patients (Table 2).

**Table 2:** Correlational analysis of serum and CSF semaphorin levels in patients with RRMS.

Semaphorines	Serum	CSF	Correlation Serum vs. CSF	
Sema3A	3.64 ± 2.40, 2.99 (0.72–11.84)	6.11 ± 1.03, 6.42 (2.94–7.78)	r p	0.019 0.915
Sema3F	7.67 ± 3.84, 6.44 (3.85–20.94)	13.19 ± 2.64, 12.96 (8.03–18.45)	r p	−0.042 0.809
Sema4A	9.11 ± 4.46, 7.94 (5.17–29.79)	10.84 ± 2.19, 10.64 (6.91–17.42)	r p	−0.256 0.131
Sema4D	12.46 ± 4.82, 10.66 (6.82–27.63)	12.87 ± 3.97, 13.55(3.10–19.95)	r p	0.183 0.278
Sema7A	6.12 ± 2.61, 5.24 (3.80–17.31)	8.54 ± 2.05, 8.56 (4.65–12.27)	r p	0.122 0.485

Mean ± SD (min–max). RRMS, relapsing–remitting multiple sclerosis; CSF, cerebrospinal fluid; Sema3A, semaphorin 3A; Sema3F, semaphorin 3F; Sema4A, semaphorin 4A; Sema4D, semaphorin 4D; Sema7A, semaphorin 7A.

Most demographic and clinical characteristics were not significantly correlated with semaphorin levels in RRMS patients. However, MoCA and SDMT scores were positively correlated with serum levels of Sema4A and Sema7A. In exploratory correlation analyses, periventricular lesion count was negatively correlated with serum Sema4A, whereas infratentorial lesion count was positively correlated with CSF Sema4A (Table 3).

**Table 3:** Correlation analysis of semaphorin levels with clinical, laboratory, and radiological characteristics.

Clinical, laboratory and radiological findings		*p*	SERUM					CSF				
			Sema3A	Sema3F	Sema4A	Sema4D	Sema7A	Sema3A	Sema3F	Sema4A	Sema4D	Sema7A
Clinical	EDSS	r p	0.069 0.683	−0.047 0.785	0.208 0.224	0.046 0.785	0.112 0.523	0.001 0.995	−0.013 0.938	0.183 0.259	0.044 0.787	0.111 0.494
	MoCA	r p	0.380 0.061	0.289 0.170	**0.510** **0.009**	0.316 0.124	**0.553** **0.005**	0.118 0.567	0.005 0.980	0.059 0.767	−0.045 0.821	−0.047 0.811
	SDMT	r p	0.234 0.261	0.278 0.188	**0.477** **0.016**	0.241 0.245	**0.460** **0.024**	0.199 0.330	0.044 0.823	−0.051 0.796	−0.217 0.267	−0.190 0.332
Laboratory, CSF	Protein	r p	−0.238 0.157	−0.229 0.178	−0.186 0.278	−0.171 0.311	−0.311 0.069	−0.088 0.604	−0.178 0.271	−0.282 0.078	−0.032 0.845	−0.099 0.543
	Cell	r p	0.057 0.736	−0.025 0.886	0.076 0.662	0.119 0.483	−0.0234 0.175	−0.033 0.848	−0.092 0.572	0.034 0.837	−0.071 0.663	0.196 0.225
	Opening pressure	r p	0.211 0.323	0.109 0.619	0.091 0.679	0.148 0.491	0.019 0.932	−0.093 0.652	−0.097 0.630	−0.223 0.263	0.182 0.364	0.054 0.788
	CSF/serum chlorine ratio	r p	−0.095 0.793	−0.097 0.574	0.063 0.717	0.063 0.698	0.122 0.452	−0.200 0.234	−0.165 0.308	−0.190 0.240	0.063 0.698	0.122 0.452
	CSF/Serum glucose ratio	r p	0.005 0.977	0.028 0.876	0.136 0.452	0.150 0.397	0.158 0.387	−0.093 0.599	−0.145 0.391	−0.144 0.395	0.176 0.297	0.203 0.228
	OCB count	r p	−0.378 0.075	−0.351 0.109	**−0.474** **0.022**	−0.328 0.127	**−0.525** **0.012**	−0.059 0.789	−0.045 0.830	0.068 0.746	0.111 0.599	**0.421** **0.036**
	IgG index	r p	0.196 0.246	0.033 0.849	0.155 0.367	0.304 0.068	0.125 0.473	−0.101 0.552	−0.206 0.201	0.049 0.765	0.233 0.148	0.256 0.111
Radiological, lesion count	Cotical/ juxtacortical	r p	0.058 0.735	−0.255 0.133	−0.120 0.485	−0.017 0.922	−0.054 0.758	−0.119 0.482	0.104 0.522	−0.122 0.455	−0.030 0.856	0.159 0.328
	Periventricular	r p	−0.291 0.080	−0.244 0.151	**−0.339** **0.043**	−0.171 0.311	−0.196 0.258	−0.070 0.681	0.075 0.646	0.188 0.245	0.052 0.750	0.261 0.104
	Infratentorial	r p	−0.124 0.464	−0.082 0.635	−0.316 0.060	−0.095 0.576	−0.216 0.213	−0.120 0.480	0.123 0.451	**0.388** **0.013**	0.095 0.559	0.210 0.193
	Spinal cord	r p	−0.086 0.613	−0.281 0.097	−0.310 0.066	−0.109 0.522	−0.149 0.392	−0.015 0.928	0.095 0.561	0.095 0.558	0.179 0.268	0.119 0.464

RRMS, relapsing–remitting multiple sclerosis; CSF, cerebrospinal fluid; EDSS, Expanded Disability Status Scale; MoCA, Montreal Cognitive Assessment; SDMT, Symbol Digit Modalities Test; OCB, oligoclonal band; IgG, immunoglobulin G. Correlation analyses were considered exploratory, and unadjusted *p*-values are presented. Bold values indicate nominal *p* < 0.05 and should be interpreted as hypothesis-generating because of the large number of comparisons.

**Figure 2:**
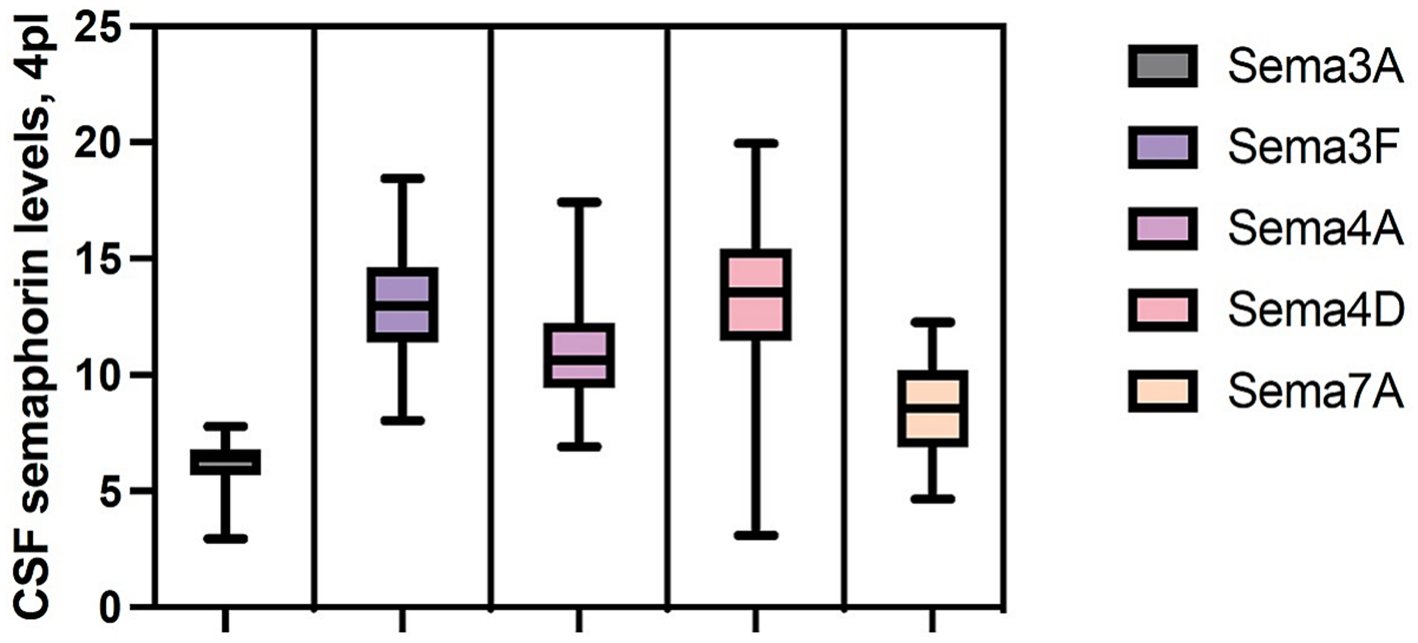
CSF levels of semaphorins in patients with RRMS. Box plots show the median and range (min–max). RRMS, relapsing–remitting multiple sclerosis; CSF, cerebrospinal fluid.

**Figure 3:**
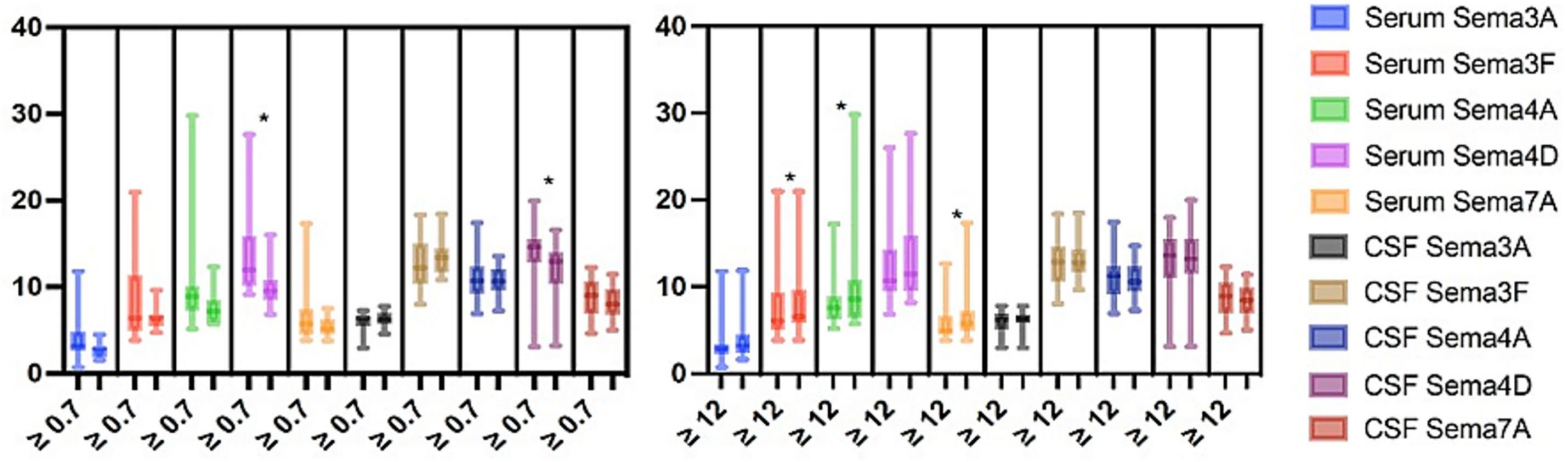
Differences in serum and CSF semaphorin levels according to IgG index (≥0.7 vs. <0.7) and CSF oligoclonal band count (≥12 vs. <12). The left panel shows comparisons according to IgG index, and the right panel shows comparisons according to OCB count. Data are presented as median (min–max). *p* < 0.05. CSF, cerebrospinal fluid; OCB, oligoclonal band; IgG, immunoglobulin G.

Among CSF semaphorins, Sema3A showed the lowest levels and Sema4D the highest levels (Figure 2). There was no significant association between OCB type and serum or CSF semaphorin levels. OCB count was negatively correlated with serum Sema4A and serum Sema7A levels, and positively correlated with CSF Sema7A levels. Serum Sema3F, Sema4A, and Sema7A levels were lower in patients with an OCB count ≥12 than in those with an OCB count <12 (*p* = 0.034, *p* = 0.013, and *p* = 0.011, respectively). Serum and CSF Sema4D levels were higher in patients with an IgG index ≥0.7 than in those with an IgG index <0.7 (*p* = 0.006 and *p* = 0.023, respectively) (Tables 4, 5 and Figure 3). Regarding radiological features, serum Sema4A levels were lower, and CSF Sema4A levels were higher in patients with infratentorial lesions than in those without infratentorial lesions (unadjusted *p* = 0.049 and *p* = 0.045, respectively; Tables 4, 5).

**Table 4:** Differences in semaphorin levels according to clinical, laboratory, and radiological characteristics.

Clinical, laboratory and radiological findings		SERUM					CSF				
		Sema3A	Sema3F	Sema4A	Sema4D	Sema7A	Sema3A	Sema3F	Sema4A	Sema4D	Sema7A
Clinical	Optic neuritis	0.806	0.685	0.761	0.589	0.472	0.666	0.711	1.000	0.776	0.820
	Motor	0.256	0.220	0.261	0.635	0.679	0.271	0.353	0.207	0.689	0.835
	Sensory	1.000	0.584	0.293	0.869	0.732	0.729	0.882	0.968	0.527	0.757
	Brain stem/cerebellar	0.909	0.520	0.741	0.316	0.725	0.402	0.827	0.850	0.373	0.358
	Other	0.222	0.513	0.452	0.332	0.545	0.046^*^	0.099	0.307	0.079	0.899
	Number of symptoms (mono-multi)	0.775	0.443	1.000	0.480	0.567	0.142	0.314	0.799	0.091	0.925
	Poor prognostic initial symptom^**^	0.518	0.104	0.181	0.845	0.909	0.408	0.739	0.989	0.383	0.883
	EDSS>2^***^	0.713	0.856	0.456	0.532	0.238	0.227	0.690	0.106	0.842	0.228
	Family history of MS	0.363	0.495	0.178	0.973	0.807	0.324	0.656	1.000	0.656	0.503
Laboratory, CSF	CSF/serum glucose ≥0.6 vs. <0.6	0.986	0.843	0.456	0.592	0.404	0.861	0.191	0.237	0.319	0.191
	OCB type	0.610	0.582	0.348	0.208	0.485	0.514	0.750	0.584	0.683	0.613
	OCB count ≥12 vs. <12	0.059	**0.034**	**0.013**	0.190	**0.011**	1.000	0.728	1.000	1.000	0.503
	IgG index ≥0.7 vs. <0.7	0.065	0.680	0.067	**0.006**	0.434	0.572	0.255	0.902	**0.023**	0.318
Radiological, lesion localization	Cotical/juxtacortical	0.728	0.102	0.446	0.892	0.780	0.684	0.778	0.338	0.600	0.600
	Periventricular	0.817	0.971	0.917	0.468	0.612	0.192	0.310	0.741	0.538	0.338
	Infratentorial	0.407	0.379	**0.049**	0.517	0.217	0.350	0.677	**0.045**	0.946	0.163
	Spinal cord	0.316	0.292	0.685	0.707	0.957	0.731	0.842	0.932	0.776	0.530

RRMS, relapsing–remitting multiple sclerosis; CSF, cerebrospinal fluid; EDSS, Expanded Disability Status Scale; OCB, oligoclonal band; IgG, immunoglobulin G; MS, multiple sclerosis. Bold values indicate statistically significant differences (*p* < 0.05). Subgroup comparisons were considered exploratory, and unadjusted *p*-values are presented. Bold values indicate nominal *p* < 0.05 and should be interpreted cautiously because of the risk of type I error due to multiple testing.

^*^No significant pairwise difference was observed in the subgroup analysis. ^**^ Poor prognostic initial symptoms included motor, brainstem/cerebellar, or sphincter dysfunction. ***Because only three patients had an initial EDSS ≥3, statistical analysis was not performed for this subgroup.

**Table 5:** Descriptive data of statistically significant laboratory and radiological features.

Semaphorines	OCB count		
	≥12	<12	*p*
Serum Sema3F	6.1 ± 2.03 5.61 (4.37–11.64)	7.32 ± 1.65 6.55 (4.74–9.66)	**0.034**
Serum Sema4A	7.37 ± 1.43 7.51 (5.17–10.16)	11.2 ± 6.42 9.11 (5.74–29.79)	**0.013**
Serum Sema7A	4.85 ± 0.94 4.54 (3.86–7.01)	6.91 ± 3.5 5.81 (4.42–17.31)	**0.011**

Semaphorines	IgG index		
	≥0.7	<0.7	*p*
Serum Sema4D	13.94 ± 5.48 11.99 (9.18–27.63)	10.28 ± 2.49 9.55 (6.82–16.01)	**0.006**
CSF Sema4D	13.93 ± 3.47 14.63 (3.1–19.95)	11.28 ± 4.23 12.99 (3.23–16.58)	**0.023**

Semaphorines	Infratentorial localization		
	Yes	No	*p*
Serum Sema4A	8.56 ± 5.38 7.19 (5.17–29.79)	9.72 ± 3.18 8.54 (5.94–17.17)	**0.049**
CSF Sema4A	11.43 ± 2.28 11.81 (7.26–17.42)	10.11 ± 1.89 10.39 (6.91–13.59)	**0.045**

Descriptive statistics were given as mean ± SD and median (min–max). OCB, oligoclonal band; IgG, immunoglobulin G; CSF, cerebrospinal fluid.

Only exploratory subgroup findings with nominal *p* < 0.05 are summarized (Bold values indicate statistical significance). These *p*-values are unadjusted and should be interpreted as hypothesis-generating rather than confirmatory.

**Figure 4:**
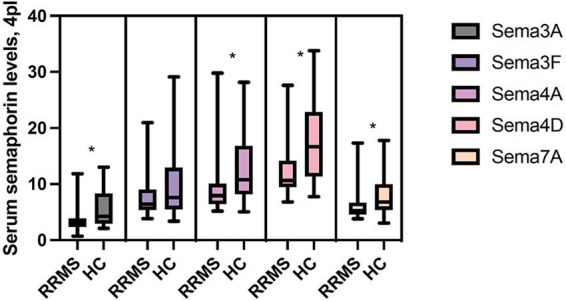
Comparison of serum levels of semaphorins between patients with RRMS and healthy controls (HC). Box plots show median values and ranges (min–max). *p* < 0.05. RRMS, relapsing–remitting multiple sclerosis; HC, healthy controls.

Serum semaphorin levels were compared between RRMS patients and healthy controls. Serum levels of Sema3A, Sema4A, Sema4D, and Sema7A were lower in RRMS patients than in healthy controls and remained statistically significant after Holm-Bonferroni correction across the five primary serum semaphorin comparisons (adjusted *p*-values of 0.005, 0.008, 0.008, and 0.048, respectively; Figure 4, Supplementary Table 4). Serum Sema3F did not differ significantly between groups (adjusted *p* = 0.165).

In ROC analyses, serum Sema3A, Sema4A, and Sema4D showed modest discriminatory ability between RRMS patients and healthy controls (Supplementary Figure 1). However, given the modest AUC values, the absence of disease controls, and the lack of an independent validation cohort, these findings should be regarded as preliminary.

## Discussion

Semaphorins are involved in the pathogenesis of immune-related diseases. The role of semaphorins in multiple sclerosis, whose pathogenesis is still unclear and for which no definitive diagnostic or prognostic biomarker has been identified, has been the subject of increasing interest in recent years (6).

By inhibiting the migration of oligodendrocyte progenitor cells into demyelinated lesions, Sema3A may play an immunomodulatory role in the pathogenesis of MS. Treatments that inhibit immune cell migration are known to significantly prevent relapses in MS. Therefore, inhibition of Sema3A/neuropilin-1/plexin-A1 may be beneficial by reducing infiltration and promoting remyelination (4). In its absence, dendritic cell migration to lymph nodes (and hence antigen-specific T-cell responses) is impaired. Its expression is reduced in serum and on immune cells in RRMS (39, 40). It has been found to be higher in patients with RRMS who are receiving injection therapy than in naive patients (41). Its decline has also been reported to have the potential as a biomarker for relapse (39). In our study, although CSF Sema3A levels were not significantly associated with the evaluated patient-related parameters, serum Sema3A levels were significantly lower in RRMS patients than in healthy controls (*p* = 0.001), in line with previous reports. This statistically significant decrease may reflect impaired peripheral immune regulation in early RRMS. However, no significant associations were found between Sema3A and other clinical or radiological parameters.

There is very little information about Sema3F in the literature. It is thought to function together with Sema3A as an axonal guidance cue during nervous system development and acts as a chemoattractant for oligodendroglial cells. Its glial and neuronal expression is upregulated in the brains of MS patients and in experimental models, suggesting a possible role in lesion remyelination (12). In contrast to Sema3A and Sema4D, Sema3F is considered to have a remyelination-promoting role (6). In our study, serum Sema3F levels did not differ significantly between RRMS patients and healthy controls. This finding may indicate that Sema3F is less strongly associated with disease onset than other semaphorins, or that its biological role is more closely related to local CNS remyelination processes than to peripheral serum changes. However, this non-significant finding should be interpreted cautiously, as the available sample size was powered mainly to detect moderate-to-large between-group differences, and modest Sema3F alterations may have remained undetected. Therefore, our findings do not exclude a possible biological role of Sema3F in RRMS. In subgroup analyses, patients with an OCB count of 12 or more had lower serum Sema3F levels, suggesting a relationship between reduced Sema3F and more pronounced intrathecal immune activity. Nevertheless, because OCB counts were assessed only in a limited subgroup of type 2 OCB patients, this association should be considered exploratory and hypothesis-generating rather than definitive.

Sema4A is elevated in serum and on the surface of dendritic cells in MS and is also higher than in neurological diseases. Elevated serum levels are associated with disease activity, the presence of Th17 cells, greater disability, and a lower response to IFN-*β* treatment (19, 40, 42–46). It has also been shown that Sema4A can be detected in the cerebrospinal fluid of MS patients and can induce oligodendrocyte cell death (47). Sema4D, implicated in immune-related diseases, also plays a role in neurodegenerative diseases such as multiple sclerosis. In EAE, it causes microglial activation, inhibition of migration, oligodendrocyte progenitor cell differentiation, and destruction of the tight junction of blood–brain barrier endothelial cells (19, 24, 29). Neuroinflammation is significantly inhibited, and the myelination process is restored when anti-Sema4D antibodies are administered (4, 24). The inhibition of this function may be a potentially valuable therapeutic target in MS (31). Very few studies of Sema4D in MS patients exist. One study highlighted that increased Sema4D levels in T lymphocytes and decreased Sema4D receptor expression in B lymphocytes may be associated with disease development in MS patients (48). In our study, serum Sema4A levels were lower in RRMS patients than in controls. In addition, serum Sema4A was negatively correlated with OCB count, periventricular lesion number, and the presence of infratentorial lesions, whereas CSF Sema4A was positively correlated with the presence and number of infratentorial lesions. These findings suggest that lower peripheral Sema4A levels may be associated with greater radiological and intrathecal inflammatory burden, while higher CSF levels in patients with infratentorial involvement may reflect compartment-specific regulation within the CNS. Although previous studies have linked Sema4A to Th differentiation, Treg stability, and inflammation, the inverse relationship between low serum Sema4A and markers of disease burden in our cohort differs from the existing literature. This discrepancy may be related to the early disease stage, treatment-naive status of our patients, or differences between peripheral and central semaphorin dynamics. We also found lower serum Sema4D levels in RRMS patients than in controls. However, Sema4D levels were higher in both serum and CSF in patients with a high IgG index at disease onset, whereas no significant correlation was found with most clinical or radiological features. This pattern may indicate that Sema4D is more closely related to intrathecal immune activation than to disability or lesion burden. Since the IgG index is a diagnostically relevant parameter in MS, the association observed in our study suggests that Sema4D may retain pathophysiological relevance despite the lower serum levels detected in the overall RRMS group. These findings may reflect inflammatory-degenerative processes at the early stage of the disease, differences in peripheral and CNS expression, or the relatively small sample size of our cohort. Given the limited and partly inconsistent clinical literature on Sema4A and Sema4D in multiple sclerosis, further studies are needed to clarify the reasons for these discrepancies. Taken together, our findings suggest that Sema4A and Sema4D may be associated with lesion distribution, compartmentalized inflammatory activity, and early intrathecal immune activation in RRMS; however, these results should be interpreted cautiously and confirmed in larger studies. Further investigations evaluating these semaphorins in serum, immune cells, and cerebrospinal fluid in treatment-naive and treated patients with RRMS and progressive MS may help clarify their biological role and provide further insight into the immunopathogenesis of MS.

In EAE, Sema7A is elevated in the CNS and the immune system, inducing inflammation, modulating the immune response, and contributing to neurodegeneration (4, 35). Sema7A deficiency has been shown experimentally to be protective and may be a valuable therapeutic target (4, 33, 36). However, in contrast to experimental studies, results from clinical trials in MS patients are conflicting. It has been reported to decrease in the cerebrospinal fluid of MS/CIS patients (49). It was reported to be lower in CSF in CIS that converted to MS, to be negatively correlated with baseline and first-year lesion counts, and to have biomarker potential for definite conversion to MS (50). In addition, there has been evidence that gene expression levels are higher in recipients of immunomodulatory therapy than in naive RRMS patients without treatment (41). In our study, as observed for several other semaphorins, serum Sema7A levels were lower in RRMS patients than in controls. In addition, serum Sema7A showed a positive correlation with cognitive test scores, suggesting that higher peripheral Sema7A levels may be associated with better preserved cognitive function. In the CSF-related immunological analyses, serum Sema7A was negatively correlated with OCB count, whereas CSF Sema7A was positively correlated; moreover, serum Sema7A levels were lower in patients with an OCB count of 12 or more. This pattern may indicate that increasing intrathecal immune activation is accompanied by a relative decrease in peripheral Sema7A and a parallel increase within the CNS compartment, consistent with compartmentalized immune responses in RRMS. Although the literature on Sema7A in MS remains conflicting, our findings suggest that decreased serum Sema7A and relatively increased CSF-associated Sema7A activity may be linked to inflammatory immune activation in early RRMS.

In our cohort, semaphorin levels were not significantly associated with sex when RRMS patients and healthy controls were analyzed separately. Likewise, no age-related differences in serum or CSF semaphorin levels were observed in either group. These findings suggest that the observed semaphorin alterations were more likely related to disease mechanisms than to demographic variables. However, given the relatively small sample size and the predominance of females in the RRMS group, subtle age- or sex-related effects cannot be completely excluded.

An important statistical consideration is the large number of biomarker-related comparisons performed in this exploratory study. Although the primary serum case–control comparisons were adjusted for multiple testing, the correlation and subgroup analyses were considered exploratory and interpreted using unadjusted *p*-values. Therefore, findings with p-values close to the conventional significance threshold should not be regarded as confirmatory. These associations may help generate hypotheses regarding compartment-specific semaphorin dynamics in RRMS, but they require validation in larger, independent cohorts with prespecified analytical endpoints.

In addition, ROC analyses suggested that serum Sema3A, Sema4A, and Sema4D may have preliminary biomarker relevance in treatment-naive RRMS. However, these findings should be interpreted cautiously, given that the discriminatory performance was modest, cerebrospinal fluid control samples were not available, and no disease control group was included. Validation in larger and independent cohorts, including appropriate disease controls, is required before any diagnostic applicability can be considered.

## Conclusion

Studies investigating immune semaphorins in multiple sclerosis remain limited. In line with previous studies, our findings suggest that low serum Sema3A levels may be associated with RRMS and may have preliminary biomarker relevance. For Sema3F, no significant difference was observed in the primary serum comparison between RRMS patients and healthy controls; however, given the limited sample size and exploratory subgroup findings regarding OCB count, a modest or compartment-specific association cannot be excluded. Regarding Sema7A, for which variable results have been reported in the literature, our findings suggest that decreased serum levels and increased CSF levels may be related to inflammatory immune activation. On the other hand, the low serum levels of Sema4A and Sema4D, which differ from previous reports, may be partly due to limitations of our study, including its small sample size and exploratory design. In this context, further studies, including larger cohorts, CSF analyses, and appropriate control groups, are needed to clarify the biological and clinical relevance of semaphorins in MS. Our results may guide future studies investigating the relationship between semaphorins and MS.

### Strengths and limitations

The strengths of this study include the simultaneous evaluation of serum and cerebrospinal fluid semaphorin levels in treatment-naive patients with relapsing–remitting multiple sclerosis, together with the combined assessment of clinical, cognitive, radiological, and immunological parameters. In addition, the inclusion of an age- and sex-matched healthy control group for serum comparisons allowed the identification of disease-related peripheral semaphorin alterations.

The main limitations of this study are the relatively small sample size, the single-center design, and the absence of cerebrospinal fluid (CSF) data in the healthy control group, which precluded direct intrathecal comparisons of semaphorin levels between patients and controls. Although the female predominance in our cohort is consistent with the known epidemiology of relapsing–remitting multiple sclerosis, the relatively small number of male patients may have limited the ability to detect sex-specific effects. In addition, because oligoclonal band (OCB) counts were assessed only in type 2 patients, the corresponding analyses were based on a limited subgroup.

No formal *a priori* power calculation was performed, which represents an important limitation of the study. The sample size was determined by the feasibility of recruiting consecutive, newly diagnosed, treatment-naive RRMS patients with available paired serum and CSF samples. Sensitivity-based considerations indicate that the present study was adequately powered mainly for moderate-to-large effects, whereas modest correlations or small between-group differences may have remained undetected. Therefore, non-significant findings, particularly those related to Sema3F and subgroup-based analyses, should not be interpreted as evidence of no association. These findings should be regarded as exploratory and require confirmation in larger, adequately powered, multicenter cohorts.

Future studies should include larger, multicenter cohorts, as well as age- and sex-matched control CSF samples obtained in appropriate clinical settings. Further analyses of serum, immune cell, and CSF semaphorins in treatment-naive and on-treatment relapsing–remitting MS and progressive MS patients are also needed to clarify the biological and clinical relevance of semaphorins in MS.

## Data Availability

The datasets generated and/or analyzed during the current study are not publicly available due to ethical and privacy restrictions. Data are available from the corresponding author upon reasonable request and subject to approval by the relevant ethics committee and applicable data protection regulations.

## Supplementary material

SUPPLEMENTARY FIGURE 1 Diagnostic biomarker potential analyses of serum semaphorins. **(A)** Sema3A (AUC: 0.722, sensitivity: 72.97%, specificity: 63.64%, *p* = 0.0003), **(B)** Sema3F (AUC: 0.596, sensitivity: 66.67%, specificity: 58.82%, *p* = 0.1633), **(C)** Sema4A (AUC: 0.721, sensitivity: %83.33, specificity: %60.61, *p* = 0.0005), **(D)** Sema4D (AUC: 0.713 sensitivity: %91.89, specificity: %52.94, *p* = 0.0009), **(E)** Sema7A (AUC: 0.665 sensitivity: %77.14, specificity: %51.72, *p* = 0.0182).

SUPPLEMENTARY TABLE 1 Comparison of serum and CSF semaphorin levels in RRMS and the healthy control group according to sex (mean ± sd, median (min–max)).

SUPPLEMENTARY TABLE 2 Comparison of serum and CSF semaphorin levels in RRMS and the healthy control group according to age (mean ± sd, median (min–max)).

SUPPLEMENTARY TABLE 3 Changes in semaphorin levels according to time of symptom onset in RRMS patients (mean ± sd, median (min–max)).

SUPPLEMENTARY TABLE 4 Differences in serum semaphorin levels between RRMS and the healthy control group (mean ± sd, median (min–max)).
