# Supplementary material for: Differences in immune-related semaphorin levels in the CSF and serum of newly diagnosed, treatment-naive patients with relapsing–remitting multiple sclerosis: a case–control study
Source: Front Neurol. 2026 Jul 14;17:1851383. doi: 10.3389/fneur.2026.1851383 (PMC13411455; doi:10.3389/fneur.2026.1851383)

**SUPPLEMENTARY TABLE 1**

| Serum | RRMS | | | | HC | | | |
| --- | --- | --- | --- | --- | --- | --- | --- | --- |
|  | Female | Male | Total | P | Female | Male | Total | P |
| Sema3A | 3.47±2.19,  2.99(0.72-11.84) | 4.18±3.06,  2.88(2.53-11.77) | 3.64±2.40,  2.99(0.72-11.84) | 0.821 | 6.08±3.70,  4.51(2.47-13.02) | 5.46±3.55,  4.20(2.12-11.63) | 5.95±3.63,  4.26(2.12  13.02) | 0.450 |
| Sema3F | 7.62±3.48,  6.55(3.85-19.88) | 7.83±5.02,  6.33(4.88-20.94) | 7.67±3.84,  6.44(3.85-20.94) | 0.774 | 9.39±6.04,  7.53(3.39-29.12) | 11.31±6.60,  10.37(4.00-2.43) | 9.84±6.13,  7.57(3.39  29.12) | 0.510 |
| Sema4A | 9.38±4.97,  7.94(5.17-29.79) | 8.13±1.55,  8.07(5.97-10.16 | 9.11±4.46,  7.94(5.17-29.79) | 0.985 | 13.13±6.27,  11.28(5.54-28.16) | 13.74±7.46,  10.51(5.06-23.63) | 13.26±6.42,  10.77(5.06  28.16) | 0.983 |
| Sema4D | 12.46±4.77,  10.72(6.82-27.63) | 12.45±5.27,  10.66 (8.67-  25.98 | 12.46±4.82,  10.66(6.82-7.63) | 1.000 | 19.5±8.14,  18.44(8.28-  33.79) | 13.94±7.53,  11.51(7.76-29.18) | 18.19±8.25,  16.57(7.76  33.79) | 0.077 |
| Sema7A | 6.46±2.88,  5.77(3.80-17.31) | 5.00±0.68,  5.01(3.86-5.95 | 6.12±2.61,  5.24(3.80-17.31) | 0.252 | 8.08±3.23,  7.34(4.22-17.79) | 7.22±4.89,  5.20(3.05-17.45) | 7.85±3.69,  6.76(3.05  17.79) | 0.126 |

| CSF | RRMS | | |
| --- | --- | --- | --- |
|  | Female | Male | P |
| Sema3A | 6.04±1.09,  6.19(2.94-7.78) | 6.38±0.74,  6.62(4.72-7.00 | 0.335 |
| Sema3F | 13.59±2.61,  13.35(9.64-18.45) | 11.81±2.39,  11.72(8.03-16.30 | 0.080 |
| Sema4A | 11.16±2.09,  11.23(7.19-17.42) | 9.73±2.28,  9.52(6.91-13.55 | 0.114 |
| Sema4D | 12.75±4.34,  13.87(3.10-19.95) | 13.28±2.39,  12.74(10.26-17.91 | 0.545 |
| Sema7A | 8.47±1.99,  8.32(4.65-12.27) | 8.77±2.35,  9.78(5.01-11.47 | 0.610 |

RRMS: relapsing-remitting multiple sclerosis; HC: healthy controls; CSF: Cerebrospinal Fluid

**SUPPLEMENTARY TABLE 2**

| Serum | RRMS | | | HC | | |
| --- | --- | --- | --- | --- | --- | --- |
|  | <40 years | >40 years | P | <40 years | >40 years | P |
| Sema3A | 3.96±2.63,  2.99(1.84-11.84) | 2.66±1.04,  2.88(0.72-3.96) | 0.270 | 5.66 ±3.41,  4.2(2.12 - 12.95) | 6.85 ±4.37,  6.2(2.47 - 13.02) | 0.789 |
| Sema3F | 7.94±4.13,  6.47(4.37-20.94) | 6.87±2.85,  6.11(3.85-11.64) | 0.450 | 9.57 ±6.31,  7.57(3.39 - 29.12) | 10.71 ±5.8,  8.27(4.96 - 19.99) | 0.537 |
| Sema4A | 9.70±4.95,  8.49(5.43-29.79) | 7.33±1.53,  7.08(5.17-10.13) | 0.117 | 12.52 ±5.76,  10.51(5.06 - 24.64) | 15.57 ±8.16,  15.07(5.54 - 28.16) | 0.352 |
| Sema4D | 12.90±5.23,  10.72(8.15-27.63) | 11.09±3.05,  10.66(6.82-15.90) | 0.453 | 19.66 ±8.55,  18.44(7.76 - 33.79) | 13.4 ±5.02,  11.39 (8.28 - 20.6) | 0.059 |
| Sema7A | 6.33±2.85  5.77(3.80-17.31) | 5.44±1.53  4.83(4.36-8.68) | 0.323 | 8.07 ±3.86  6.85(3.05 - 17.79) | 7.27 ±3.35,  6.21(4.22 - 13.01) | 0.549 |

| CSF | RRMS | | |
| --- | --- | --- | --- |
|  | <40 years | >40 years | P |
| Sema3A | 6.16±0.87,  6.45(4.03-7.34) | 5.96±1.46,  6.10(2.94-7.78) | 0.614 |
| Sema3F | 13.52±2.85,  13.23(8.03-18.45) | 12.20±1.57,  12.19(9.74-14.61) | 0.209 |
| Sema4A | 10.93±2.33,  10.60(6.91-17.42) | 10.55±1.79,  11.16(7.19-12.32) | 0.747 |
| Sema4D | 12.89±4.15,  13.43(3.10-19.95) | 12.82±3.56,  13.68(3.48-15.41) | 0.890 |
| Sema7A | 8.50±2.14,  8.85(4.65-11.47) | 8.66±1.84,  8.08(6.31-12.27) | 1 |

RRMS: relapsing-remitting multiple sclerosis; HC: healthy controls; CSF: Cerebrospinal Fluid

**SUPPLEMENTARY TABLE 3**

|  | | <1 month  n=12 | 1 month-1 year  n=18 | >1 year  =10 | P |
| --- | --- | --- | --- | --- | --- |
| Serum | Sema3A | 3,8 ± 2,9  3 (1,9 - 11,8) | 3,3 ± 1,6  2,9 (1,6 - 7,9) | 4,1 ± 3,2  2,9 (0,7 - 11,8) | 0.772 |
|  | Sema3F | 7,1 ± 1,7  6,5 (5,5 - 11,2) | 7,6 ± 3,9  6 (3,8 - 19,9) | 8,5 ± 5,3  6,5 (4,4 - 20,9) | 0.694 |
|  | Sema4A | 10,6 ± 7,1  8,1 (5,7 - 29,8) | 8,7 ± 3,3  8 (5,9 - 17,2) | 8 ± 2  7,8 (5,2 - 10,2) | 0.787 |
|  | Sema4D | 12,7 ± 5,7  10,5 (8,7 - 27,6) | 11,7 ± 4,2  10,5 (6,8 - 25) | 13,7 ± 5,1  11,9 (9,2 - 26) | 0.772 |
|  | Sema7A | 6,7 ± 3,8  5,7 (4,6 - 17,3) | 6 ± 2,2  5 (3,8 - 12,6) | 5,7 ± 1,4  5,8 (3,9 - 7,9) | 0.867 |
| CSF | Sema3A | 6,1 ± 0,9  6,2 (4 - 7,3) | 6,2 ± 1,2  6,5 (2,9 - 7,8) | 5,9 ± 0,9  6,1 (4,6 - 7) | 0.386 |
|  | Sema3F | 14,6 ± 2,9  13,9 (9,6 - 18,5) | 12,9 ± 2,2  13 (9,7 - 18,3) | 12 ± 2,6  11,6 (8 - 16,3) | 0.868 |
|  | Sema4A | 11,5 ± 1,9  11,5 (7,3 - 14,7) | 10,3 ± 1,7  10,4 (7,2 - 13,5) | 10,9 ± 3,1  11,1 (6,9 - 17,4) | 0.843 |
|  | Sema4D | 13 ± 4,1  13,1 (3,2 - 20) | 12,2 ± 4,6  13,9 (3,1 - 17,9) | 14 ± 2  14,4 (11 - 16,7) | 0.495 |
|  | Sema7A | 9,1 ± 2,3  9,8 (5 - 11,5) | 7,9 ± 1,8  7,9 (4,6 - 11,3) | 9 ± 2,2  9,3 (5 - 12,3) | 0.275 |

CSF: Cerebrospinal Fluid

**SUPPLEMENTARY TABLE 4**

|  | RRMS | HC | Unadjusted p | Holm-Bonferroni adjusted p |
| --- | --- | --- | --- | --- |
| Sema3A | 3.64 ± 2.39  2.99 (0.72- 11.84) | 5.95 ± 3.63  4.26 (2.12-13.02) | **0.001** | **0.005** |
| Sema3F | 7.67 ± 3.84  6.44 (3.85-20.94) | 9.84 ± 6.13  7.57 (3.39-29.12) | 0.165 | 0.165 |
| Sema4A | 9.11 ± 4.45  7.94 (5.17-29.79) | 13.26 ± 6.42  10.77 (5.06-28.16) | **0.002** | **0.008** |
| Sema4D | 12.46 ± 4.81  10.66 (6.82-27.63) | 18.19 ± 8.25  16.57 (7.76-33.79) | **0.002** | **0.008** |
| Sema7A | 6.12 ± 2.61  5.24 (3.80-17.31) | 7.85 ± 3.69  6.76 (3.05-17.79) | **0.024** | **0.048** |

RRMS: relapsing-remitting multiple sclerosis; HC: healthy controls. Unadjusted p-values are shown together with Holm-Bonferroni adjusted p-values for the five primary serum case-control comparisons.

**SUPPLEMENTARY FIGURE 1**

A
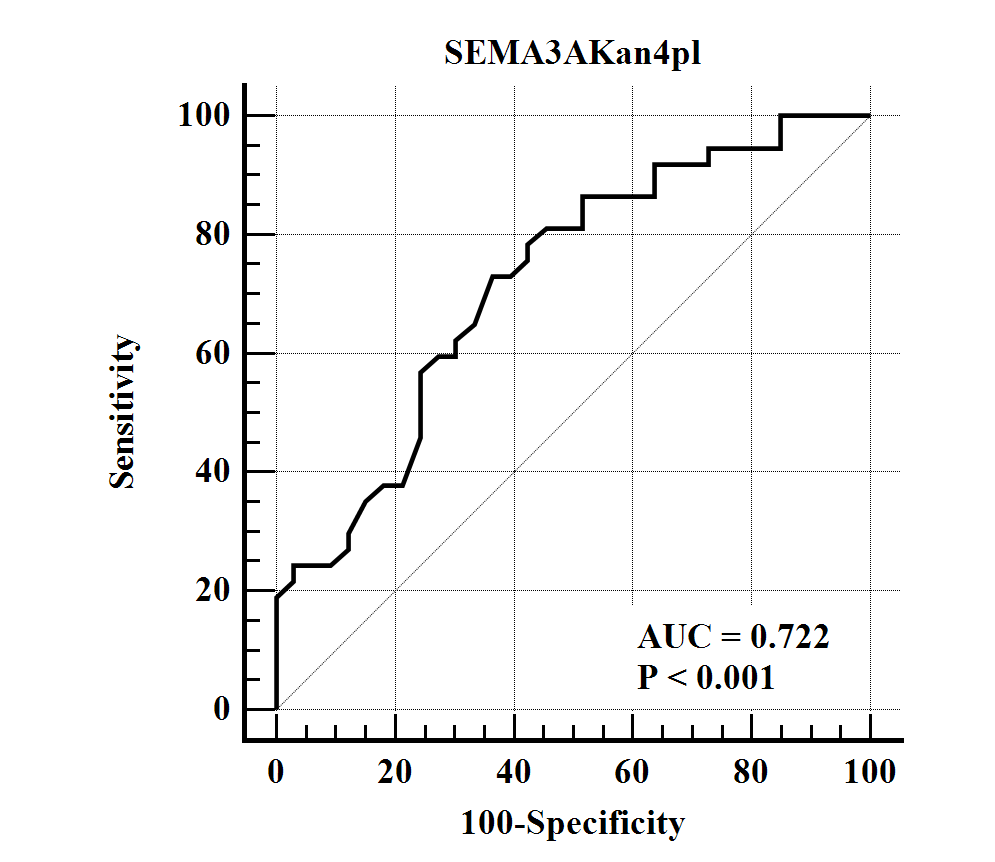
 B
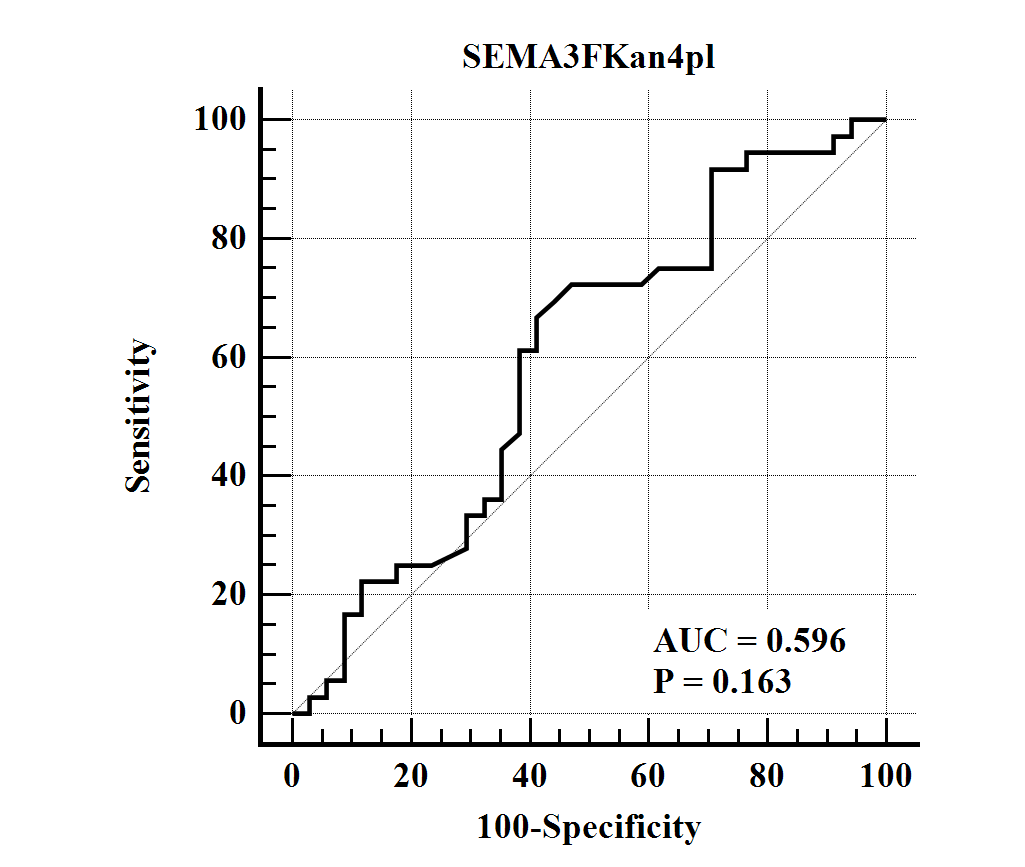


C
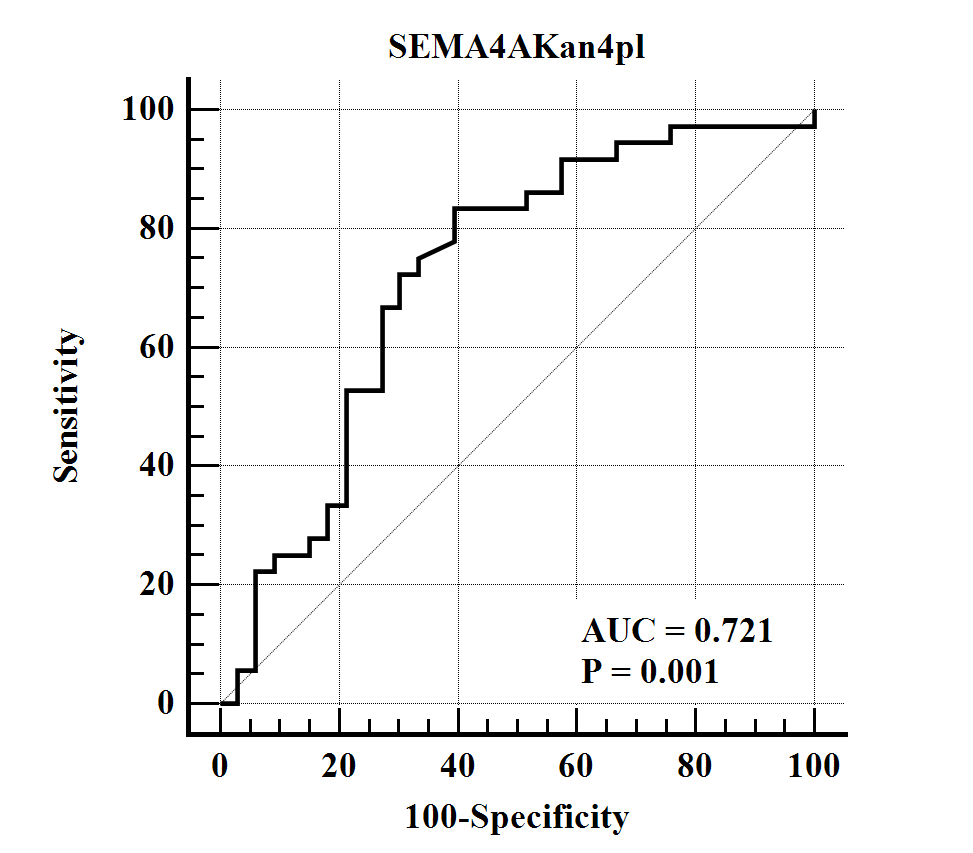
 D
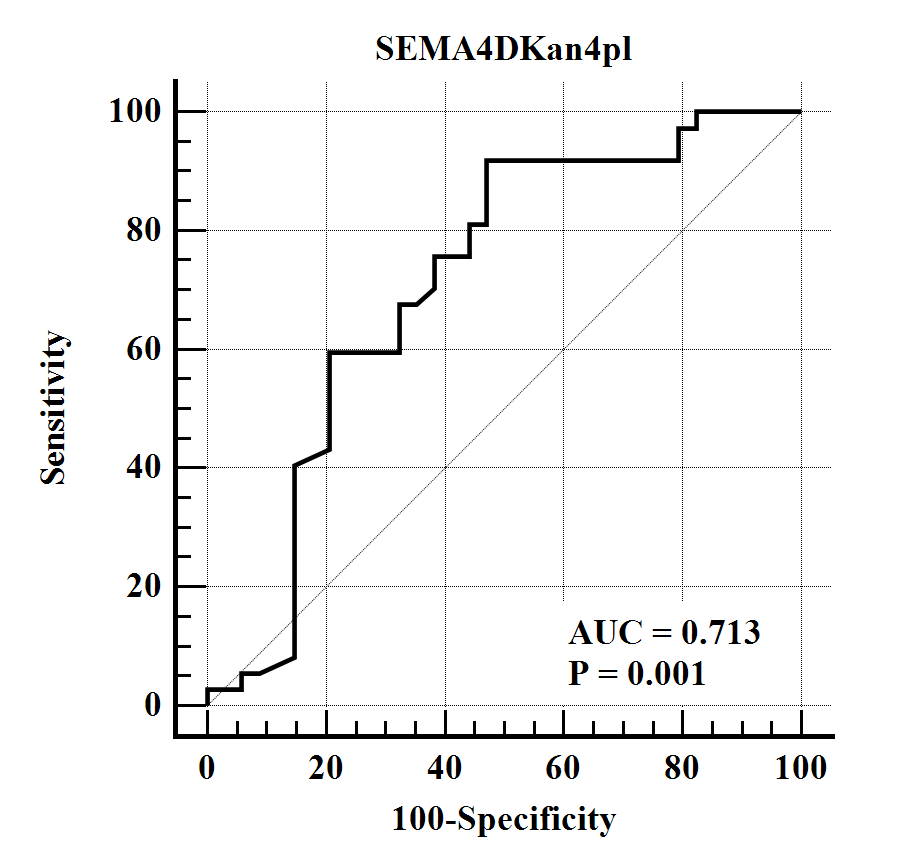


E
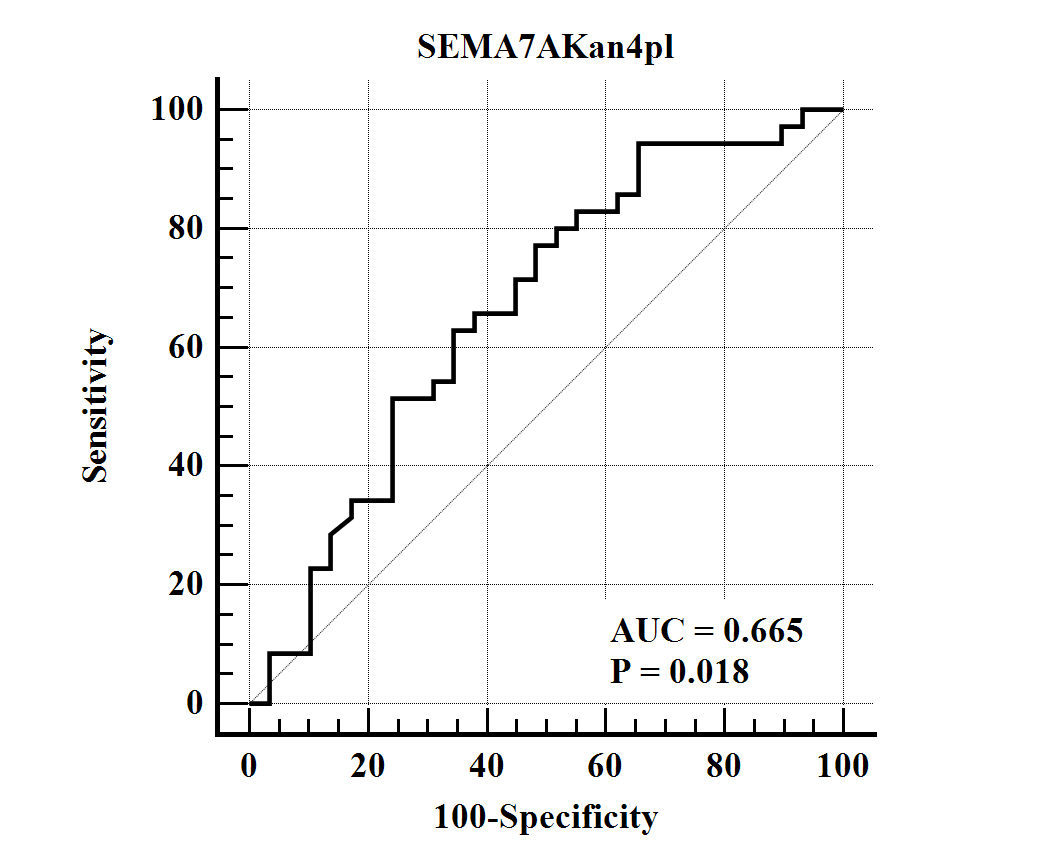

Supplement: Supplementary file 1 [file Supplementary_file_1.docx]
